# Protein-based antigen presentation platforms for nanoparticle vaccines

**DOI:** 10.1038/s41541-021-00330-7

**Published:** 2021-05-13

**Authors:** Brian Nguyen, Niraj H. Tolia

**Affiliations:** Laboratory of Malaria Immunology and Vaccinology, National Institute of Allergy and Infectious Diseases, National Institute of Health, Bethesda, MD USA

**Keywords:** Biotechnology, Microbiology, Vaccines, Immunology

## Abstract

Modern vaccine design has sought a minimalization approach, moving to the isolation of antigens from pathogens that invoke a strong neutralizing immune response. This approach has created safer vaccines but may limit vaccine efficacy due to poor immunogenicity. To combat global diseases such as COVID-19, malaria, and AIDS there is a clear urgency for more effective next-generation vaccines. One approach to improve the immunogenicity of vaccines is the use of nanoparticle platforms that present a repetitive array of antigen on its surface. This technology has been shown to improve antigen presenting cell uptake, lymph node trafficking, and B-cell activation through increased avidity and particle size. With a focus on design, we summarize natural platforms, methods of antigen attachment, and advancements in generating self-assembly that have led to new engineered platforms. We further examine critical parameters that will direct the usage and development of more effective platforms.

## Introduction

**Fig. 1:**
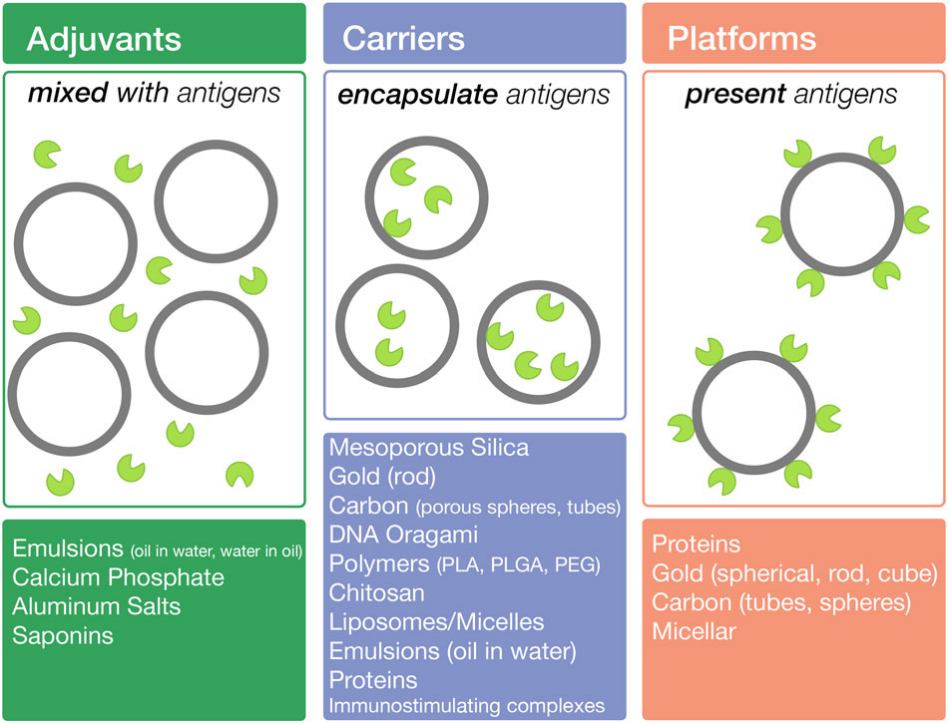
The three categories of nanoparticle roles in vaccines as adjuvants, carriers, and platforms with the descriptions of the role below. Gray circles represent nanoparticles while, green circular indented units represent antigens. Abbreviations PLA, PLGA, and PEG correspond to polylactic acid, polylactic-co-glycolic acid, and polyethylene glycol, respectively. Modified and adapted from Zhao et al.^2^.

The term “nanoparticle” has varied usage in the scientific literature. For biological products the Unites States Food and Drug Administration (FDA) classifies a nanoparticle as a material or substance that has been deliberately manipulated to have dimensions between 1 to 100 nm or up to 1000 nm if it exhibits physical, chemical, or biological effects dependent on its size^1^. Particles in this size range can significantly influence biological systems, driving the expansion of nanoparticle research in biology and medicine. In vaccinology, nanoparticles serve three major roles: as adjuvants, carriers, or presentation platforms, determined by how the vaccine antigen interacts with the nanoparticle (Fig. 1)^2^. Upon vaccination, a nanoparticle is used to improve the immune response through one or a combination of these roles. Nanoparticles have demonstrated remarkable success as particulate adjuvants^2,3^ and nucleic acid delivery carriers^4^, however, this review will specifically focus on protein-based nanoparticle platforms. Previous publications have summarized the characteristics, utilization, and efficacy of nanoparticle platforms in vaccines^2,5–8^ and the bioengineering strategies^9–13^ of self-assembling proteins applicable for the design of new potential platforms. This review serves to provide an updated, comprehensive and concise analysis on current platforms, the design strategies for novel platforms, and how the structural characteristics of these platforms affect the immune response at the molecular level.

### Nanoparticle platforms in vaccine development

Nanoparticle platforms involve the attachment of antigen to the surface of a particle, either inorganic or organic in nature, (Fig. 1) to promote an immune response through enhanced trafficking and recognition by cellular receptors. Inorganic platforms raise concerns of toxicity and non-biodegradability. Micellar platforms, which have been used to present SARS-CoV-2 Spike trimers, may be limited to antigens with a transmembrane domain^14^. In contrast, protein-based platforms are highly biocompatible, can assemble homogenously, and can be effectively tailored to suit any antigen. Protein nanoparticles injected intravenously have been shown to freely travel through circulatory and lymph vessels with rapid accumulation in the thyroid and spleen, advantageous for establishing humoral immunity^15^. Furthermore, protein-based platforms enable antigen attachment through genetic fusion or affinity tags complexes, which allows for a homogenous decoration of antigens on the platform. The first protein platforms utilized virus capsid proteins isolated from their infectious components and exploited their naturally oligomeric nature to form stable nanoparticles called virus-like-particles or VLPs^5^. Subsequently, many naturally oligomeric proteins such as ferritin, lumazine synthase, and C4b-binding protein (C4bp) orthologs have been developed for platform design. These platforms vary in size, ranging from as little as 4 nm with C4-binding protein-like particles to as large as 120 nm with Influenza M1 VLPs^6^ (Table 1). Recent advances in computational protein design have enabled development of synthetic platforms, where pre-existing proteins are engineered to assemble into highly oligomeric complexes, which rival naturally occurring platforms in size and antigen valency.

**Table 1:** Structural comparison of different self-assembling proteins; size was measured from the longest axis while # subunits indicate the number of monomers within the nanoparticle. Fused subunits are counted as one monomer.

Name	Size (nM)	MW (kDa)	# Subunits	PDB	References
IMX313	4.4	150	7	4B0F	^45^
Nsp10	8.4	204	12	2G9T	^72^
T3 (10)	11	276	12	4DCL	^77^
T32 (28)	11	571.8	24	4NWN	^78^
Ferritin	12	456	24	1AEW	^64^
T33 (15)	12.2	406.8	24	4NWO	^78^
O3 (33)	13	478.8	24	3VCD	^77^
Lumazine Synthase	15	1002.6	60	1HQK	^69^
T3 + 2	16	602.64	12	3VDX	^76^
O3 + 4CC	18	886	24	n/a	^80^
HbsAg VLP	22	3076.9~	96~	n/a	^50^
O3 + 2	22.5	750	24	n/a	^79^
E2p	23.2	1595.8	60	1B5S	^71^
I3 (01)	25	1432.8	60	1VLW*	^82^
I3 + 5CC	25	2167.8	60	n/a	^81^
I52 (32)	25.3	1993.8	120	5IM4	^83^
AP205 VLP	27.2	2520	180	5LQP	^59,60^
I53 (50)	27.5	2479.2	120	EMD-0350 (Cryo-EM)	^83^
I32 (28)	28.6	2053.2	120	5IM6	^83^
Ico532-1	30	2520	60	n/a	^84^
M1 VLP	120	~	~	n/a	^6^

Asterisk (*) indicates that the engineered nanoparticle structure was not available and a PDB structure of the original building block was provided instead. Tilde (~) indicates that value may vary due to lipid composition.

### Protein nanoparticle platforms improve the immune response to antigens in vaccines

The effectiveness of a prophylactic vaccine is determined by the generation of a long-lasting T-cell-dependent IgG antibody response^16^. Generating this response involves the activation of T-cells by antigen presenting cells (APC) followed by the activation of B-cells by antigen and a specialized T helper cell, the T Follicular helper (T_FH_)^17^. APCs phagocytize, process, and present antigens to T-cells, which if recognized, become activated to differentiate into T helper cells. Naive B-cells, which require two signals to mature into high-affinity IgG plasma cells, receive the first signal from the crosslinking of multiple B-cell receptors (BCR) with an antigen that has directly entered the lymph node or was presented by APCs, followed by endocytosis of the receptor and antigen, and then presentation of the processed antigen on the surface. The second signal is delivered upon recognition of the B-cell-presented antigen with a T_FH_ cell, which activates the B-cell to mature into an IgG-producing plasma cell.

Many successful vaccines in the past consisted of attenuated vaccines that were highly effective but were potentially infectious. This deficiency prompted the creation of inactivated components or subunit vaccines, for example those used in modern influenza vaccines. Subunit vaccines typically have excellent safety profiles by consisting of isolated antigens necessary to establish an immune response, and as a result may also be less immunogenic. However, vaccination with certain subunit vaccines such as those against Human papillomavirus (HPV), Cervarix and Gardasil, has seen remarkable success in protection^18^. This success has been attributed in part to the ability of the antigen, the major capsid protein, to self-assemble into highly oligomeric spherical VLPs. However, many antigens, such as those used in influenza subunit vaccines, do not self-assemble into nanoparticles. In these cases, self-assembly can be generated by attachment of these antigens to an oligomeric protein platform. Studies have shown that scaffolded antigen induce stronger and longer lasting neutralizing antibody titers, as well as greater protection^19–24^.

**Fig. 2:**
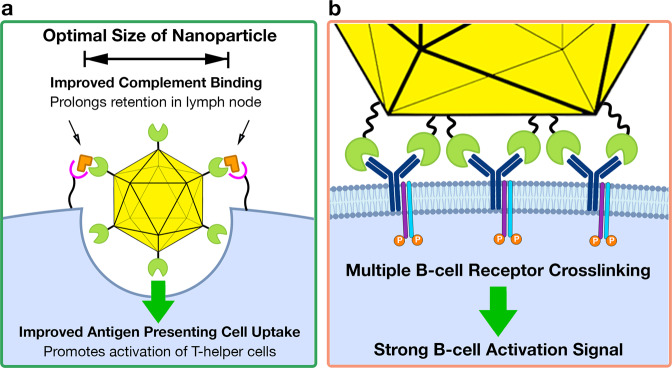
Advantages of protein nanoparticle vaccines. Yellow icosahedrons represent a protein nanoparticle platform while green circular sectors represent a genetically fused antigen. **a** Beneficial effects of increased size by presenting antigen on a nanoparticle platform. One of these effects is improved binding of complement indicated by the rectangular orange shape, on the surface of the nanoparticle. The bound complement facilitates binding to complement receptors on APCs such as follicular dendritic cells and promotes retention of the opsonized nanoparticles in the lymph nodes. Another effect of increased size is enhanced uptake of nanoparticles by APCs, indicated by the circular cavity and direction of travel arrow into the APC, in light blue. **b** Enhanced B-cell activation through the interaction of multiple antigens with BCRs, which are embedded within the membrane of the B-cell.

Two characteristics of nanoparticle platforms contribute to generating the B-cell IgG response: (1) the attachment of the antigen to a larger scaffold, which improves APC uptake and retention in lymph follicles and (2) the repetitive array of antigens, which enables efficient binding and activation of multiple B-cell receptors (Fig. 2). Attachment of antigens on particles increases the overall particulate size into an optimal size range for efficient uptake by APCs, which allows for greater presentation of antigen by APCs to activate T-helper cells^25,26^ (Fig. 2a). Larger particles are also efficiently opsonized with complement^27^. Opsonization promotes binding to the surface of FDCs (follicular dendritic cells), elongating retention in lymph follicles, and enhancing antigen presentation to B-cells^27^. Particles displaying numerous antigens can then facilitate B-cell activation through efficient crosslinking with multiple BCRs^26,28^ (Fig. 2b). Evidence of this claim is demonstrated by one study that examined how the density of an antigen, a model peptide, affected memory immune response^29^. High-density conjugation of an antigen to a VLP activated a specific IgG antibody response, while low-density conjugation did not (despite increased antigen quantity), suggesting an effect outside the total amount of antigen. In addition to enhanced B-cell activation caused by repetitive antigen surfaces, the immune system appears to be trained to recognize these surfaces, such as viral capsids and bacterial pili, as a non-self indicator^28,30^. Because of this effect, protein platforms have enabled self-antigen, that are normally dispersed, to break self-tolerance and mount an immune response^31^. The ability to break self-tolerance has facilitated the use of protein platforms in cancer vaccine development through the presentation of tumor-associated antigens (TAAs), which are self-antigens overexpressed in cancerous tissue but rarely observed in normal tissue^31^.

Protein nanoparticles can help in the development of vaccines for immuno-evasive pathogens such as human immunodeficiency virus (HIV), influenza, and malaria^5^. Antibody-dependent-enhancement (ADE), which contributes to the infection of HIV and Dengue, occurs when non-neutralizing antibodies bind and encourage infection of immune cells^32^. To counter this possibility a technique called epitope focusing has been used to design antigens that direct an antibody response to neutralizing epitopes. This technique that involves isolating neutralizing epitopes from antigens are oftentimes poorly immunogenic in isolation. However, when attached to a nanoparticle platform, the increased presentation allows these antigens to yield a strong yet targeted humoral response^33^. In addition to epitope focusing, T-cell epitopes introduced in the interior of the platform can be used to activate a cell-mediated immune response^34,35^. Incorporation of universal CD4 + T-cell epitopes can help establish a humoral response against the antigen through recruitment of helper-T cells while CD8 + T-cell epitopes can generate killer T-cells against a specific pathogen. The combination of these techniques may enable successful vaccination against difficult pathogens.

### Antigen attachment

**Fig. 3:**
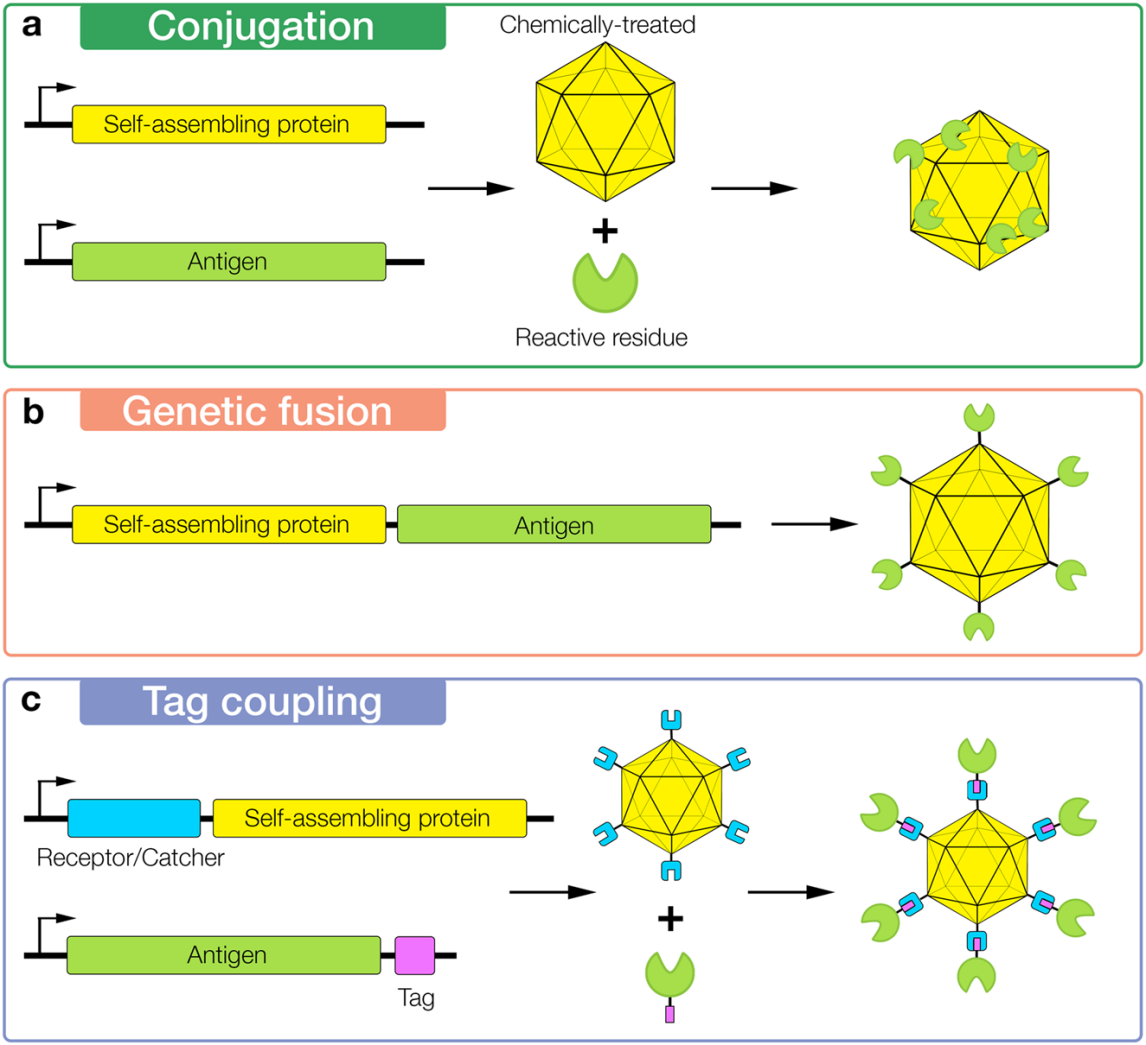
Methods of Antigen attachment to nanoparticle platform. Protein sequences are indicated by rectangular boxes, arranged left to right from N to C terminals. Protein nanoparticle platforms are represented by a yellow icosahedron and antigens are represented as a green circular sector. **a** A typical chemical conjugation scheme for protein nanoparticles, which involves the chemical treatment of a reactive amino acid on either the nanoparticle or antigen to conjugate with a reactive amino acid on the other respective protein. In this case, the nanoparticle is chemically treated to bind with a reactive amino acid residue on the antigen. **b** Genetic fusion with the platform, indicated by a distinct black line between the antigen and nanoparticle. **c** An example of tag-coupling systems, where the genetic fusion of a protein receptor or protein catcher, represented as cyan colored squares with a cavity, to one component allows for binding to another component through a genetically fused tag, represented as a small pink rectangle.

Three different methods are used to attach antigens for presentation on nanoparticles: chemical conjugation, genetic fusion, and tag coupling (Fig. 3). These methods allow platforms to be decorated with numerous antigens, resulting in increased presentation and size. Conjugation relies on chemical treatment to crosslink antigens to the platform, which can lead to an uneven decoration. The other methods, such as genetic fusion and tag coupling, attach antigen specifically to the terminals and offer a more precise antigen arrangement.

In a typical chemical conjugation used for antigen attachment to VLPs, the protein nanoparticle undergoes chemical treatment to crosslink surface-exposed lysines to engineered or surface-exposed cysteines of a target antigen^36^ (Fig. 3a). This method has seen success in licensed *Haemophilus influenzae* type b (Hib) vaccines^37^, which conjugates polysaccharides of Hib to an immunogenic protein platform such as Tetanus toxoid. The established nature of this method has led researchers to explore vaccines that use conjugation to attach protein-based antigen. Decoration of *Pseudomonas aeruginosa* exoprotein A (EPA), an inherently immunogenic protein, with a malaria antigen has led to a 75- to 110-fold increase in specific antibody generation versus immunization without EPA^38–40^. For this case the antigen was lysine-treated and conjugated to the cysteine of EPA^41^. Unlike other methods, conjugation can be used to decorate monomeric proteins with numerous antigens. However, the uneven decoration of this method may lead to poor display of antigen and inefficient B-cell activation against neutralizing epitopes^42^. Nevertheless, chemical conjugation has been proven to significantly improve immune responses despite these supposed limitations.

Genetic fusion and tag coupling can often provide greater precision through site-specific antigen attachment. Genetic fusion of the antigen to the nanoparticle platform is the most direct method (Fig. 3b) if the appropriate terminal on the nanoparticle, depending on the antigen’s own terminals, is accessible and properly orients the antigen. However, there are some cases where separate expression and purification of the antigen and platform may be preferred, such as when fused constructs do not express properly or inhibit proper folding of the nanoparticle. Tag-coupling systems allow for independent expression and modular attachment of antigens, although they still require some genetic fusion (Fig. 3c). Most tag-coupling scenarios involve the binding of a tag, which is fused to a terminus on one protein, to a protein receptor or catcher, which is fused to the partner protein. Popular tag-coupling systems include Biotin-Avidin, HaloTag, and SpyTag/SpyCatcher^36^.

SpyTag/SpyCatcher has had the most extensive use in antigen presentation platforms^20,42–44^. This system uses *Streptococcus pyogenes* fibronectin-binding protein FbaB, which has been split in two components, SpyCatcher (113 aa) and SpyTag (13 aa). Nanoparticle platforms that are fused to a SpyCatcher form an irreversible peptide bond with the SpyTag fused to the antigen or vice versa and can be fused to either the N- or C-terminal. Attachment of two different antigens can be accomplished by combining SpyTag/SpyCatcher with another tag-coupling system such as SnoopTag/SnoopCatcher^45^. Tag coupling can result in a lower numbers of antigen per particle than genetic fusion, due to inefficient coupling^46^. In addition, the genetic fusion of a large protein (e.g., SpyCatcher) to the nanoparticle may interfere with the attachment of antigen. Ultimately, tag-coupling systems allow for rapid modular attachment of different antigens to a nanoparticle platform, at the cost of introducing more components.

### Natural nanoparticle platforms: virus-like particles (VLPs)

Virus-like particles (VLPs) are composed of self-assembling viral envelope or capsid proteins, ranging from 20 nm to 800 nm in diameter (Table 1), which are devoid of any infectious component^2^. VLPs were the first nanoparticles to be used in vaccines due to the relative ease of design that exploited naturally high stability and self-assembly. VLPs have seen extensive use in clinical trials and commercially approved vaccines as platforms and demonstrate a reliable proof-of-principle for protein platform technology.

The world’s first licensed and approved malaria vaccine, RTS,S, is based on VLPs^47^. RTS,S uses the hepatitis B virus surface antigen small-envelope protein (HbsAg) to self-assemble into a spherical nanoparticle^48^. HbsAg, which is the main component in currently approved hepatitis B vaccines^49^, forms the lipid viral envelope that encloses the capsid and is able to form noninfectious particles 22 nm in diameter^50^. Further structural characterizations suggests that an HbsAg VLP consists of 24 tetramers, with a rhombicuboctahedron-like shape, although exact composition may vary due to lipid nature^51^. Antigen attachment to the envelope in RTS,S is accomplished by genetic fusion of a malaria antigen to the N-terminus of HbsAg^48^. The fused HbsAg is then coexpressed with unmodified HbsAg to allow for nanoparticle formation, although nanoparticles consisting solely of fused HbsAg have also been created^52^. Vaccination with RTS,S confers moderate protection against malaria, and attachment of the antigen to the VLP has been credited with significantly increasing its efficacy^53^.

Influenza Matrix 1 (M1) protein-based VLP is a second enveloped VLP platform, which has seen usage in clinical trials for an influenza vaccine^54^. This platform forms large spheroid particles around 120 nm in diameter^55^. To assemble into VLPs M1 must interact with proteins containing a cytoplasmic tail region such as HA (hemagglutinin) and NA (neuraminidase)^56^. M1 VLPs have been made with different combinations of influenza surface glycoproteins or envelope proteins from other viruses and has been expressed in mammalian and insect systems^57,58^. M1 VLPs were used in a SARS-CoV vaccine, consisting of M1 envelope protein and chimeric spike protein^19^. Replacement of the spike protein transmembrane and C-terminus regions with those of HA allowed the antigen to insert itself into the lipid membrane formed by the envelope protein. Vaccination of mice with the chimeric spike VLP protected mice against SARS-CoV. Comparatively, vaccination with spike alone required the addition of adjuvant for protection.

**Fig. 4:**
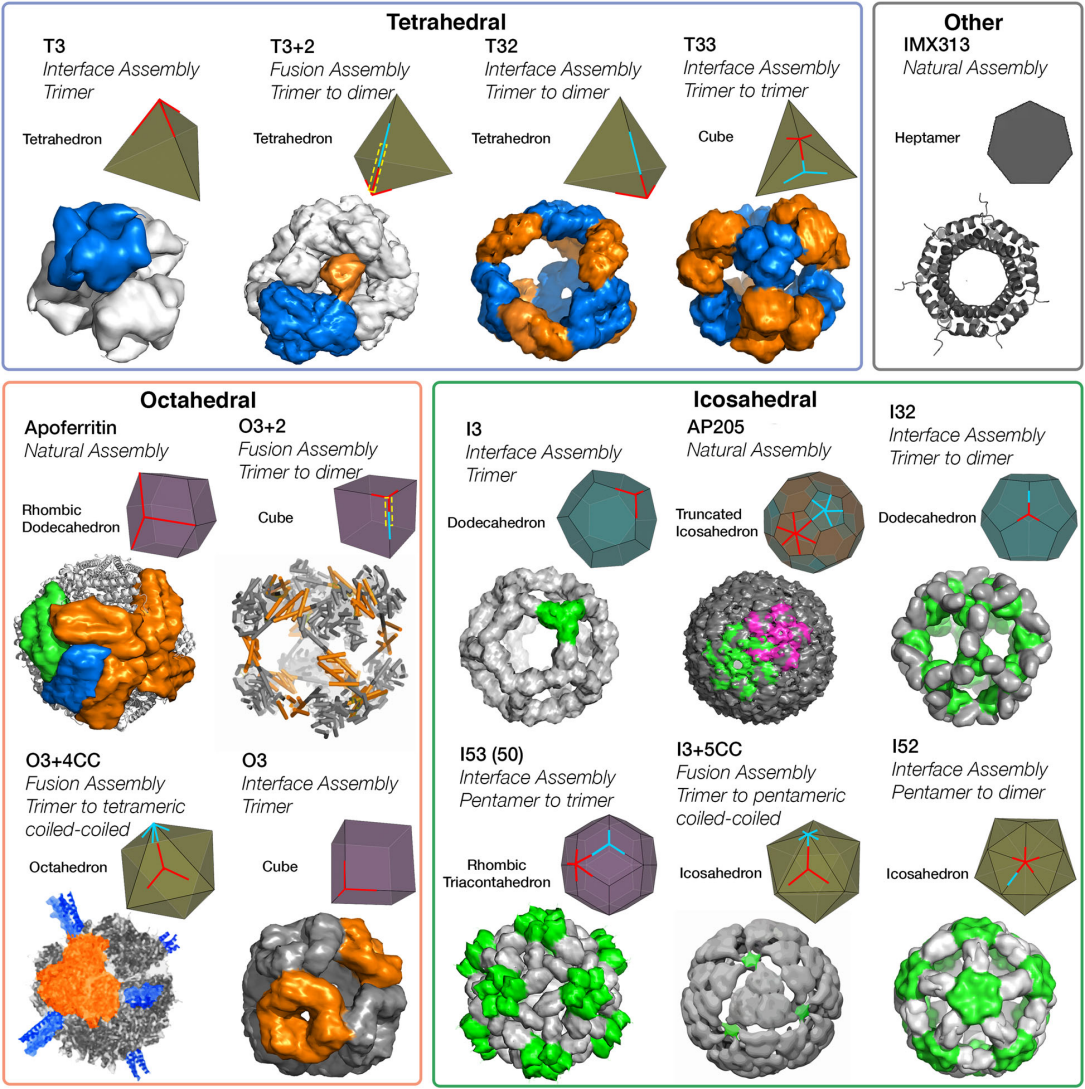
Summary of published engineered self-assembling nanoparticles and naturally assembling nanoparticles, sorted by symmetry type. For the names of engineered nanoparticles, the first letter indicates symmetry of the design T, O, or I for tetrahedral, octahedral, icosahedral, respectively. The first number indicates the oligomeric state of the primary building block, while the second number represents the oligomeric state of the secondary building block, if present in the design. The abbreviation “CC” stands for a coiled-coil motif. A plus (+) between the first and second number indicates that self-assembly is generated by genetic fusion between two monomers of the primary and secondary building block, versus Interface design with Rosetta. As a visual aid, the primary building block (red) and secondary (cyan) are aligned to the vertices or faces of a polyhedra^98,99^ that resembles the geometry of the nanoparticle. Genetically fused building blocks are emphasized with a yellow-dashed box.

Capsid VLPs based on bacteriophage AP205 coat protein have shown relative ease of antigen attachment^59^. In contrast to enveloped VLPs that incorporate a lipid membrane, capsid-based VLPs are completely composed of protein. 180 monomers of the coat protein oligomerize to form a capsid that resembles a truncated icosahedron with 20 hexameric facets and 12 pentameric vertices (Fig. 4)^60^. The coat protein exists primarily as dimers and five dimers form the pentameric vertices while six dimers form the hexameric facets. The dimers are interwoven between adjacent pentameric vertices or hexameric facets. The N- and C-termini of the coat protein are located close to the threefold axis of the hexameric facets, allowing for ideal attachment of trimeric antigens to either terminus. Attachment of a host self-antigen to AP205 elicited a highly immunogenic response that overcame B-cell unresponsiveness to self-antigens^59^, demonstrating the effectiveness of nanoparticle presentation. In another study, a lysine and cysteine-rich Inter-Domain Region (CIDR) antigen from *P. falciparum* erythrocyte membrane protein 1 (PfEMP1) was attached to AP205 with the Spytag-Spycatcher coupling system^20^. AP205 coupled with CIDR produced significantly higher antibody titers after immunization in mice versus non-coupled AP205 & antigen or antigen alone. As a cancer vaccine, AP205 elicited an auto-antibody response against human epidermal growth factor receptor-2 through genetic fusion to the AP205 VLP^61^.

VLPs have demonstrated remarkable improvements in improving the humoral immune response when used as a platform for antigens. However, VLPs can be difficult to produce due to low expression yields and the presence of host cell contaminants from expression systems^62^. VLPs assembled in vivo can encapsulate host DNA and other host proteins, potentially due to their nature, which encourages the packaging of genomic information. Removal of such contaminants involves complex purification or in vitro disassembly and reassembly steps^62^. VLPs also suffer from stability issues, possibly due to a lack of viral genome^63^. Additionally, enveloped VLPs require eukaryotic host expression systems in order to acquire their lipid membrane. VLPs are well-established, however, manufacturing complications hinder their adoption as platforms.

### Nanoparticle platforms: non-VLP self-associating proteins

As an alternative to VLPs, non-viral proteins that are highly oligomeric have been used as platforms. These platforms are often enzymes or homeostasis proteins that can be easily produced. A hallmark of self-associating proteins is a single protein component that readily assemble into stable highly oligomeric structures that complement icosahedral VLPs.

A popular non-viral platform is Ferritin, a protein involved in intracellular iron storage that is found in nearly all organisms and consists of 24 monomers with a molecular weight of 18 kDa each^64^. The complex consists of 8 trimers with octahedral symmetry and resembles a rhombic dodecahedron: a polyhedron with both threefold and fourfold symmetry^64^ (Fig. 4). The close proximity of the N-termini to the threefold axis allows for easy attachment of trimeric antigens. In contrast, the C-terminus is buried and unavailable for antigen presentation. Mammalian expression systems have been the standard for ferritin-based vaccines, although *E. coli* expression and refolding has also been examined^65,66^. *Helicobacter pylori* ferritin has been commonly used in vaccine design due to its sequence divergence from human ferritin; when fused to HA, *H. pylori* ferritin elicited antibody protection against influenza with greater potency than commercial influenza vaccines^67^. A two-component dual-antigen ferritin derived from *Trichoplusia ni* has also been produced, and elicited neutralizing antibodies against both targets^68^. A recent study has successfully presented SARS-CoV-2 trimers fused with a C-terminal SpyCatcher on ferritin with a N-terminal SpyTag using a mammalian secretion expression system^46^. Addition of N-linked glycosylation sites to the nanoparticle improved expression yield. Successful presentation of trimers was indicated by binding to SARS-CoV-2 antibodies. Ferritin is a robust and well-established platform that provides a natural alternative to VLPs. However, ferritin is also significantly smaller and contains less subunits than VLPs (Table 1).

Lumazine synthase (LS), an enzyme involved in riboflavin synthesis, is an icosahedral non-VLP platform that has significantly more subunits than ferritin. Sixty monomers of pentameric protein naturally assemble into particles resembling a dodecahedron^69^. Both N- and C-termini of LS are surface-exposed and contain threefold and fivefold symmetry, although the N-terminus appears to be closer to the threefold apex than the C-terminus. The proximity of the termini to the symmetry axis may stabilize the presentation of trimeric or pentameric antigens. Genetic fusion of a trimeric HIV surface antigen (gp120), which was truncated and stabilized with a coiled-coil linker to the C-terminus of LS from *Aquifex aeolicus* significantly improved B-cell activation compared to antigen alone^21^. Similar to ferritin, LS has also successfully presented SARS-CoV-2 trimers on LS with a similar construct design, glycosylation modifications, and expression system used for ferritin^46^. LS is typically expressed in a mammalian cell secretion system, although *E. coli* expression has also been successfully utilized for LS^70^.

An alternate icosahedral non-VLP platform is dihydrolipoyl acetyltransferase (E2p), an enzyme that assembles into a 60-mer hollow dodecahedron from 20 trimers and forms the core of the pyruvate dehydrogenase complex^71^. The E2p nanoparticle is slightly larger than LS (Table 1) and is able to present larger trimeric HIV surface antigens, such as full-length gp120 or gp140, through genetic fusion^22^. Attachment of the trimeric antigen to the nanoparticle increased B-cell stimulation compared to antigen alone. Antigens are fused at the N-terminus, which is located near the apex of the trimer, making it ideal for trimeric antigens, whereas the C-terminus (which is located at the dimeric interface between the trimers) is inaccessible.

The nonstructural protein 10 (nsp10) has recently gained interest as a nanoparticle due to its presence within the coronavirus genus, including the SARS-CoV-2 virus^72^. Nsp10 consists of 12 monomers that assemble into a spherical particle resembling a dodecamer with twelve faces. Both N- and C-terminals are surface-exposed and are located on separate threefold axes, which allows for the attachment of antigen, especially trimeric, to both terminals. Genetic fusion of nsp10 with a γ-herpesvirus antigen, gD, elicited binding antibody titers in rabbits^72^.

Smaller non-spherical particles have also been utilized. IMX313, a *Gallus gallus* ortholog of C4bp, forms a heptameric ring-like protein^73^. The malaria antigen Pfs25 was fused to the N-terminal of IMX313, expressed in yeast, with vaccination resulting in significantly increased antibody titers compared to vaccination with the antigen alone^73^. A dual component, dual antigen IMX313 was also successfully developed through genetic fusion of SpyCatcher at the N-terminus and SnoopCatcher at the C-terminus, which was expressed in *E. coli* with refolding. The genetic fusions enabled attachments of two distinct malarial antigens Pfs25 and Pfs28 to IMX313 through coupling with SpyTag and Snooptag, respectively^45^. The multimeric antigens produced a significantly stronger antibody response than monomeric antigens.

Many highly oligomeric proteins have been successfully used as platforms. These proteins have distinct arrangements than their capsid VLP counterparts enabling unique nanoparticle presentation and design. Platforms such as Ferritin, LS, and E2p also contain a trimeric threefold favorable for the attachment of trimeric viral antigens such as the Spike protein of SARS-CoV-2. However, most of these self-assembling platforms are smaller in size than VLPs and may have only one terminus available for antigen attachment, which can limit the valency and compatibility for antigens.

### Engineered nanoparticle platforms

Through rational or computational design, dimeric, trimeric, tetrameric, or pentameric proteins have been engineered to assemble into larger, highly oligomeric complexes that offer greater control over antigen stoichiometry, spacing, and particle size. These engineered particles present additional platforms for vaccines beyond the limited number of natural platforms. In order to develop novel platforms several methods and techniques of generating self-assembly have been developed. There are several design parameters for designing self-assembling proteins for use as a platform. First, the geometric symmetry and shape of the desired nanoparticle are determined by type of building blocks used. Second, self-assembly must be promoted by either fusing two different building blocks or engineering an interface between building blocks. Lastly, at least one terminal should be exposed and accessible for antigen attachment. Described below are the design and evolution of novel self-assembling proteins that have led to engineered platforms.

### Coiled-coil-based protein design

Self-assembling peptide nanoparticles (SAPNs) are large protein assemblies composed of numerous coiled-coil domains. SAPNs are able to oligomerize into large icosahedral complexes because of interactions between individual coiled-coil domains. Formation of these complexes has been accomplished through the genetic fusion of a trimeric coiled-coil to a pentameric coiled-coil^34^, and the utilization of a trimeric coiled-coil that binds to a dimeric coiled-coil^74^. SAPNs are limited by the relatively small surface area of the monomeric building blocks. It is difficult to fuse large proteins to the monomers without them interfering with assembly, thus limiting the use of SAPNs to display small peptides or epitopes.

### Tetrahedral protein design

Tetrahedral protein scaffolds were among the first to be explored^75^ and optimized in 2012^76^. Producing a tetrahedral platform requires the oligomerization of four trimeric building blocks (Fig. 4). One way of achieving this oligomerization is by engineering a dimeric interaction between two monomers of two separate trimers. The four trimers act as the vertices of a tetrahedron while the dimeric interactions serve to link the vertices (Fig. 4). In the **T3** + **2**^76^ platform, oligomerization was achieved through a genetic fusion between a monomer of a trimer to a monomer of a dimer. This fusion allowed for the trimeric building blocks to self-assemble through the use of interconnecting dimers. Advances in the protein engineering software Rosetta^77,78^ allowed for the creation of a single component tetrahedron, **T3**^77,78^. By introducing a dimeric interface on the primary trimeric building block, the team removed the need for fusing a dimeric building block. The **T32**^78^ design is similar to the **T3** + **2** design but with a engineered dimeric interface between the primary and secondary building blocks. In the **T33** design^78^, four primary trimers act as the vertices of the tetrahedron and are joined together by secondary trimers aligned on the faces (Fig. 4), resulting in a two-component tetrahedral and octahedral nanoparticle.

### Octahedral protein design

For octahedral designs, self-assembly is achieved through the linking of 8, primarily trimeric, building blocks with dimeric interactions. The **O3** design satisfied this criterion by using 8 trimeric building blocks with designed dimeric interfaces^77^. Alternately, the genetic fusion of a trimer to a dimer using an alpha-helical linker resulted in an **O3** + **2** design^79^. Both strategies produce cube-shaped nanoparticles, however, the fusion strategy led to polydisperse assemblies consisting of possible tetrahedrons and triangular prisms caused by the different possible symmetries that 3-folds with 2-folds can assemble into. A rigid-alpha linker was utilized to restrict the angle between the trimer and the dimer to drive octahedral assembly^79^.

Although coiled-coil particles are limited to peptides, hybrid platforms developed may circumvent this limitation^80,81^. Specifically, in the **O3** + **4CC** design, a trimeric protein used as the primary building block was fused to a tetrameric coiled coil, generating octahedron-like particles with tight packing between the trimeric faces and no discernable cavity, as seen on other engineered platforms due to the small secondary building block^80^. An important design component was the linker length and flexibility between the primary and secondary building block and the most successful **O3** + **4CC** design produced octahedral assemblies with a homogeneity of 73.3%, determined by Analytical Ultracentrifugation^80^.

### Icosahedral protein design

In order to generate icosahedral self-assembly, either 20 trimeric vertices or 12 pentameric vertices must be linked together. One of the first icosahedral platforms, the **I3**, fulfills the first method^82^. It utilized a trimeric building block with an engineered dimeric interface to link the trimeric vertices together. The twenty trimers assemble to form a dodecahedron, with the trimeric building blocks aligned to the threefold vertices. Several two-component icosahedral platforms have been subsequently developed. The **I32**, notably, forms a dodecahedron similar to the **I3** platform but with a secondary dimeric building block inserted between the two trimers^83^. By changing the primary building block to a pentameric one in an **I52** model, a platform resembling an icosahedron was created, with the pentameric building block aligning to the fivefold vertex and the secondary dimeric building blocks aligned to the edges^83^. **I53** designs with a pentameric primary building block and a secondary trimeric building block were further developed^83^. In this design, the pentameric building blocks were aligned with the vertices of an icosahedron, while the trimeric building blocks were aligned with the triangular faces to create assemblies resembling a rhombic triacontahedron (Fig. 4). Utilizing the same design template as the I53 platform an icosahedral hybrid coiled-coil fusion platform, **I3** + **5CC** using a coiled-coil as the pentameric building block was produced^81^. The tight packing between the triangular faces causes the design to resemble an icosahedron rather than a rhombic triacontahedron. The triple component fusion design, Ico532, consists of a trimeric coiled coil fused with a flexible linker to a dimeric building block, which was rigidly fused to a pentameric building block^84^. 60 monomers of the fusion protein assemble to form a structure resembling an Icosahedron, similar to the I52 (Fig. 4) structure but with the triangular cavities filled by the trimeric coiled-coils.

The design of self-assembling proteins has evolved from smaller, simpler, tetrahedral complexes to icosahedral complexes that rival the size of VLPs (Table 1). These designs contain symmetrical structures unseen in nature and yet to be explored as potential platforms. A notable difference for these novel proteins compared to natural proteins exists in their packing, with several designs (Fig. 4) featuring porous cavities, which arise due to the current limitations of generating self-assembly.

### Some assembly required

The two strategies for generating self-assembly, fusion or engineered interfaces, each have their own advantages and disadvantages. Fusion is computationally less demanding but faces issues with non-specific assemblies, though several strategies have been developed to encourage uniform assemblies. Engineering interfaces have yielded homogeneous particles that has been successfully utilized as platforms. However, the low success rate of this strategy requires intensive screening of numerous designs.

The fusion strategy is rather straightforward: two monomers with different oligomeric stoichiometries are fused together. The two oligomerization domains must be held together rigidly else they may assemble irregularly^75^. The use of a rigid alpha-helical linker connecting the helical termini of the building blocks mitigated the formation of heterologous assemblies^75,76,79^. In addition, particular angles between a trimeric and dimeric building blocks were fused with a rigid linker that favors a certain symmetry^75^. However, even with the optimized α-helical fusion, the octahedral cube **O3** + **2** has been shown to form tetrahedrons and triangular prisms^79^. The hybrid coiled-coil **O3** + **4CC** designs also required linker optimization to achieve homogeneity^80^. Initial designs utilized a trimeric primary building block fused to a dimeric coiled-coil building block through a flexible linker. This resulted in dimeric, tetrahedral, and octahedral complexes^85^. Their octahedron design resulted in a significantly homogeneous population of proper complexes^80^ while their icosahedron design resulted in a nearly homogenous population^81^. They observed that by varying the linker length between the primary and secondary building block, they were able to optimize homogeneity. The use of a fourfold and threefold in their **O3** + **4CC** and a fivefold and threefold in their **I3** + **5CC** may also restrict the oligomerization compared to less specific threefold and twofold combinations. This factor was taken into account with the Ico532 design, although some heterologous assemblies still occurred. The design also contains an available trimeric N-terminus, which may be potentially used to genetically fuse antigens. In summary, optimizing linker length, reducing flexibility, and applying symmetry-based constraints should be taken into account for fusion strategies to reduce undesired assemblies.

Engineered interfaces follow similar geometric symmetry principles to the fusion strategy, but instead of fusing two oligomeric stoichiometries together it introduces another oligomeric binding site. The specificity of the engineered interface limits undesired assemblies, promoting the use of this strategy for platform development. GFP was successfully fused to the trimeric building block of the **T33**-21 design, resulting in a nanoparticle displaying a total of 24 copies of GFP^82^. One or two GFP proteins were attached to **I3**-01 through genetic fusion at the N- and C-terminus of the self-assembling protein^82^, though the N-terminal may be more suitable for antigen attachment due to the C-terminal being buried. A 19 kDa Designed ankyrin repeat protein (DARPin) was also successfully fused to **T33**-21 using an α-helical linker^86^ resulting in the presentation of 12 copies on the nanoparticle and enabling the small protein to be visualized by Cryo-EM. The **I3**-01 nanoparticle has been fused to malarial antigens using SpyCatcher-Spytag^43^ in comparison with the AP205 VLP platform. Although **I3**-01 presented less antigen, 60 compared to 180 in AP205, they both induced similar antibody levels. The **I3**-01 design has also been genetically fused to a stabilized trimeric HIV envelope antigen gp140^23^, with a ten amino acid length linker to account for the relatively large spacing of the N-termini. An alternative linker, which incorporated a T-cell epitope was also successfully incorporated. The trimeric antigen presented on **I3**-01 generated significantly higher levels of antibodies compared to the trimeric antigen alone. Similarly, **I53**-50 has been developed as an HIV vaccine, utilizing a prefusion trimer stabilized envelope antigen called SOSIP fused to the N-terminus of the trimeric secondary building block^24^. Presentation of ConM genotype SOSIP trimers on **I53**-50 demonstrated increased neutralizing antibody titers compared to SOSIP trimers alone. However, presentation of BG505 genotype SOSIP on **I53**-50 elicited lower antibody titers and poorer neutralizing antibody response than BG505 SOSIP alone. This occurrence was attributed to less accessibility to the neutralizing epitopes of BG505, which were located at the base of the spike, due to attachment with the platform. However, further analysis demonstrated no change in antibody titers against other base-located epitopes with **I53**-50 fused and non-fused SOSIP. Ultimately, this result suggests that it may be beneficial to orient antigen with neutralizing epitopes apically exposed. **I53**-50 design has been utilized in a vaccine for respiratory syncytial virus (RSV)^87^. A prefusion-stabilized variant of the RSV F glycoprotein trimer was fused to a trimeric foldon domain located at the N-terminus of the trimeric secondary building block. Neutralizing antibody titers induced by the antigen fused to **I53** were ten times greater than those that were induced by the trimeric antigen alone. **I53-**50 has recently been utilized as a vaccine for SARS-CoV-2 with the Spike glycoprotein receptor-binding domain (RBD) genetically fused to the N-terminus of the trimeric building block with varying flexible linker lengths^88^. In mice, immunizations with the RBD fused with I53 designs generated a strong antibody response, while monomeric RBD failed to generate a detectable response, and stabilized prefusion Spike trimer (S-2P) elicited a weaker response. Similar results were seen in neutralizing antibody productions. RBD fused to **I53** was able to confer protection against SARS-CoV-2 replication while monomeric RBD and S-2P did not.

In conclusion, advancements in protein design have allowed for engineered platforms that have been successfully used in prototype vaccines. With comparable immunogenicity to natural platforms, these platforms surpass natural non-VLP platforms in size (Table 1) while having trimeric and dimeric-folds suitable for attachment of common trimeric and dimeric antigens. Furthermore, the two-component engineered-interface platforms allow for straightforward purification through controlled assembly. However, several limitations exist in the creation of engineered platforms such as heterogeneity with the fusion method and successful design yield with the engineered interface method. Strategies to alleviate heterogeneity will allow the use of fusion proteins as platforms. Improvements in structural prediction and interface design will increase the success rate of the engineered interface method and permit higher levels of packing. The development of new platforms and their design strategies will allow different configurations and a greater range of parameters to be examined and optimized for improved vaccine efficacy.

### Critical parameters to consider for platform selection and design

Several factors such as size, antigen density, and surface properties are critical for the successful implementation of nanoparticles in vaccines. These parameters are described below in an attempt to guide the usage and development of platforms for improved immunogenicity. Although discrepancies may arise due to differences in nanoparticle composition the findings highlight mechanisms such as cellular uptake, opsonization, trafficking, and B-cell activation, that can be used to evaluate the performance of a platform.

**Fig. 5:**
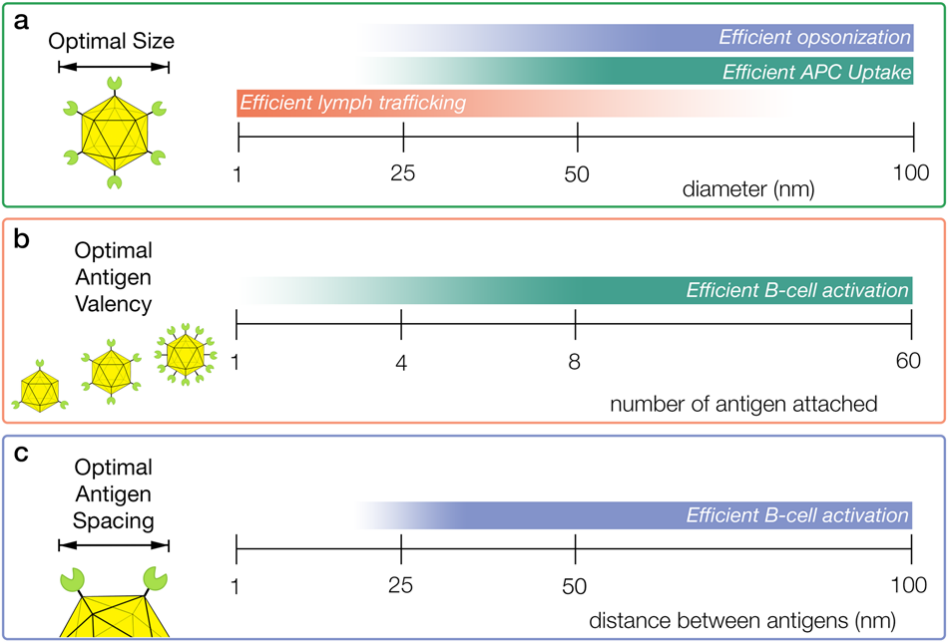
Critical parameters of nanoparticle platforms and potential optimal ranges based on current research. Gradient increases in intensity from less optimal to most optimal. **a** Parameters for optimal size of a nanoparticle with a trend as size increases for enhanced opsonization and APC uptake, but inhibited lymph node trafficking. **b** Trend for improved B-cell activation as antigen valency increases. **c** The ideal range between antigen in order to facilitate efficient B-cell activation, with potential steric constraints preventing efficient B-cell activation at distances below 28 nm.

Efficient cellular uptake, lymph follicle retention, and entry into lymph nodes are three critical factors that lead to improved immunogenicity and protection that is influenced by the size of a nanoparticle platform (Fig. 5a). Particles under 250 nm, which include most if not all protein platforms, utilize clathrin and scavenger receptor-dependent uptake pathways^25^. Testing of polystyrene particles ranging from 40 to 1500 nm in diameter determined that particles below 500 nm had the highest uptake by dendritic cells^89^. Specifically, polystyrene particles of 40, 100, and 500 nm all had relatively similar levels of uptake. Mathematical models have predicted the optimal size for cellular uptake to be between 20 and 30 nm^90^. Spherical gold particles, conjugated to albumin as an antigen, had a lymph follicle retention time of 5 weeks with particles 50 and 100 nm in diameter compared to 48 h with particles 5 and 15 nm in diameter^91^. Furthermore, the larger particles were more likely to be presented on the FDC surface, allowing greater delivery of antigen to B-cells, and higher levels of B-cell maturation. Conversely, the smaller particles were able to enter the lymph node more rapidly than the larger particles. A similar result was seen with polypropylene sulfide nanoparticles, where particles 25 nm in diameter entered the lymph nodes 10 times more efficiently than 100 nm particles^92^. Thus, the optimal platform may be around 20–50 nm, large enough for efficient uptake and retention in lymph follicles, small enough for entry into the lymph node (Fig. 5a).

Optimal B-cell activation is dependent on the antigen valency and spacing (Fig. 5b, c). One study measured how valency affected B-cell activation through comparison of the monomeric engineered HIV gp120 antigen, eOD-GT, versus protein platforms with 4, 8, or 60 copies of eOD-GT^93^. The 8-mer and 60-mer antigens demonstrated enhanced B-cell activation, B-cell proliferation and B-cell localization upon immunization of mice and this effect was not observed with lower valency antigens. Vaccination with the 60-mer also showed improved T-cell recruitment, IgG titers, and was able to rescue a low-affinity version of the antigen to induce B-cell differentiation. In contrast, examination of B-cells induced by 4-mer and 60-mer vaccinations revealed that the lower valency 4-mer had higher titers of B-cells binding to antigen probes, suggesting that lower valency antigens generated higher affinity B-cells. However, differences in the angle and orientation with which the antigen was presented or other differences due to protein composition may have impacted the display of accessible epitopes. Additionally, differences between the protein platforms such as size, flexibility, and composition may also introduce confounding factors. In order to eliminate these factors a recent study used DNA origami platforms that could present antigens with defined valency and organization^94^. The study compared valency using icosahedron shaped DNA platforms that presented 1, 2, 3, 5, 10, 30, or 60 copies of eOD-GT, with the benefits of increased valency on B-cell activation plateauing at 5. However, this result may have been hindered by inadequate antigen spacing. The study measured effects of antigen spacing between two antigens on a rod-shaped DNA platform with distances from 7 to 80 nm. B-cell activation increased as distance increased, plateaued at a distance of 28 nm and remained similarly elevated at 80 nm. The findings from this study suggest that steric constraints hinder BCR recruitment to antigen less than 30 nm apart (Fig. 5c).

Beyond size and structure other factors such as surface charge and hydrophobicity can influence the trafficking of particles and thus affect immunogenicity. Positively charged particles has been shown to promote uptake by macrophages and DCs due to electrostatic attraction to negatively charge membranes^95^. On the other hand, hydrophobic particles have been shown to promote DC uptake, possibly through enhanced cell membrane interaction^96^ or complement recruitment^26^. However, the attachment of antigens can significantly change these surface properties, and thus it may be more appropriate to evaluate these properties in context of compatibility with the antigen. Further research is needed to comprehensively analyze how the surface charge and hydrophobicity of the platform affects trafficking and immune response.

These findings suggest the parameters for the ideal platform may be 20–50 nm in diameter with antigen spaced 30 nm apart. However, a more comprehensive study containing more particles with smaller intervals in size is needed for testing of uptake, opsonization, and lymph node trafficking. Furthermore, differences in particle composition may affect the results of size as-well. A conclusive answer to the impact of valency will require testing of valencies with optimally spaced antigen. Antibody responses against the platform has been demonstrated in vaccines^97^, which may potentially divert the humoral immune response from the antigen. Continued research and testing of more platforms are needed to determine the effects of this implication, as well as evaluate parameters for the development of more optimized platforms.

### Future directions

Protein-based nanoparticles have demonstrated the ability to revolutionize vaccine development for various diseases. The design of optimal nanoparticles that effectively present antigens to produce the desired immune response is an active area of investigation. Computational design of nanoparticles has matured and allows for the expansion of available platforms for study. There are countless other possible building block arrangements that have yet to be explored as platforms. Additionally, all the engineered platforms share a common trait—a reliance on creating a dimeric interaction between the building blocks for self-assembly; more complicated symmetries may be investigated in the future. These approaches, along with more standardized methodology, are expected to contribute significantly to improving vaccines and alleviating the burden of global diseases.

## Data Availability

No data were generated for the review article.
